# Persistence and viable but non-culturable state induced by streptomycin in *Erwinia amylovora*

**DOI:** 10.3389/fmicb.2024.1346300

**Published:** 2024-02-21

**Authors:** Yeon Ju Kim, Hyun Seo Choi, Duck Hwan Park

**Affiliations:** ^1^Interdisciplinary Program in Smart Agriculture, Kangwon National University, Chuncheon-si, Republic of Korea; ^2^Plant Medicine Program, Division of Bioresource Sciences, College of Agriculture and Life Sciences, Kangwon National University, Chuncheon-si, Republic of Korea

**Keywords:** antibiotics, *Erwinia amylovora*, fire blight, persister cell, streptomycin, viable but non-culturable state

## Abstract

Persister cell and viable but non-culturable (VBNC) state of bacteria are survival strategies against antibiotics and various environmental stresses, respectively, but they tend to be ignored in agriculture fields, even though bacteria can regain their abilities to survive and produce disease once those stresses disappear. This study was carried out to determine whether persister cell and VBNC state in *Erwinia amylovora* are present after exposures to streptomycin, the length of their persistence, and the steps needed to decrease the inoculum. Persister cells were observed using biphasic killed growth curve for 4–8 h when the late stationary phase cells of *E. amylovora* were cultured in liquid medium containing streptomycin. This state was maintained for up to 12 h based on the colony forming units (CFUs) of the colonies that grew on the mannitol glutamate yeast extract (MGY) medium after streptomycin was removed. The CFUs on the MGY medium were lower than the total count determined using the LIVE/DEAD Kit, suggesting that persister cells and VBNC state might co-exist for up to 12 h after exposure to streptomycin. However, after 12 h, *E. amylovora* cells did not continue to grow on the medium for 9 days, suggesting that they entered a VBNC state at that time and remained in a persistent state. In addition, based on the Redox Sensor Green staining method, the presence of both states was confirmed for up to 12 h, and only then did the VBNC state became apparent. Furthermore, persister cells were observed for up to 24 h, and damaged cells reduced when *E. amylovora* cells were culture in distilled water with streptomycin, indicating that the uptake of lower nutrients in *E. amylovora* led to prolonged persister cells and VBNC state, which are more likely to survive after streptomycin treatments. The addition of sucrose and oxytetracycline to distilled water containing streptomycin reduced persister cells than other sources did. Thus, to inhibit the spread of fire blight, management techniques must consider the hazards of using streptomycin treatments that induce dormancy, such as persister cells and VBNC state, beyond the development of resistant strain.

## Introduction

Fire blight is caused by the Gram-negative bacterium *Erwinia amylovora*. It was first detected in 2015 (Myung et al., 2016; Park et al., 2016) and has since spread to 28 cities/districts in Republic of Korea in 2022, making it the most destructive disease in pome fruits, including apple and pear trees in Republic of Korea (Ham et al., 2020a,b; Choi et al., 2022; Jik Lee et al., 2022). Thus, chemical control strategy based on copper compounds and agricultural antibiotics was primarily adopted as a management technique against continual fire blight situation in Republic of Korea. Among the chemical control agents, agricultural antibiotics such as streptomycin, oxytetracycline, oxolinic acid, and validamycin have been registered by the Rural Development Administration of Korea (RDA) and used intensively twice during the blooming season by spraying directly into flowers (Park et al., 2017; Choi et al., 2022). However, the European Union, the United States, and Canada have prohibited the use of antibiotics because of several adverse effects such as increased disease resistance to antibiotics and subsequent public health issues and ecosystem disconnection caused by alteration of microbial diversity in soils and waters (Fried et al., 2014; USDA, 2019; Mikiciński et al., 2020). The concerned authorities in Republic of Korea is aware of these issues, and there are several policies currently in place to reduce the use of antibiotics. However, agricultural antibiotics are still used in agriculture fields across the country to treat agricultural disease, especially fire blight, because there are no alternative control agents. *E. amylovora* is a quarantine pathogen in Republic of Korea; hence, efficacy test must be conducted in small and insulated green houses. Streptomycin had been reported to show the strongest control effect among tested antibiotics (Norelli and Gilpatrick, 1982; McManus and Jones, 1994) but its effects varied depending on environmental conditions and disease severity between experimental plots (*personal communication with Dr. Lee, Y. H*. *in RDA*). Streptomycin was the first aminoglycoside antibiotic isolated in 1943 from *Streptomyces griseus* (Schatz et al., 1944) and has been used in agricultural fields since 1955 (McManus et al., 2002). Streptomycin, like many other aminoglycoside antibiotics, is also composed of an inositol derivative linked to at least one amino sugar, containing hydroxyl and amino groups, and streptamine (Cunha, 2006; Becker and Cooper, 2013). These amino sugars are the key binding elements that interact with the 16S rRNA and the ribosomal protein S12 of the 30S ribosome, interfering with initial tRNA selection in protein synthesis (Carter et al., 2000). Thus, streptomycin causes bacterial death by destabilizing membranes, inhibiting ribosomal activity, and disrupting metabolism and respiration due to lack of translational fidelity (Davis et al., 1986; Davis, 1987; Kohanski et al., 2007). Hence, its killing effect, low phytotoxicity, and cost effectiveness have made it the most widely used antibiotic against fire blight since the 1960s in USA and 2015 in Republic of Korea (Moller et al., 1981; Park et al., 2017). However, molecular mechanisms such as enzymatic inactivation (*strAB*) and spontaneous mutation (*rpsL*) have been reported as key factors of streptomycin resistance in *E. amylovora* (Chiou and Jones, 1995; Sundin and Bender, 1996), and the streptomycin resistant strains that mutated in those genes are present worldwide, including the USA, Canada, New Zealand, Israel, Lebanon, and Mexico (McManus et al., 2002; De León Door et al., 2013; Tancos et al., 2016; Sundin and Wang, 2018), suggesting that the use of streptomycin may no longer be the best strategy to control fire blight. Thus, streptomycin resistance in *E. amylovora* is seems to be a major obstacle to its use; therefore, its incidence is monitored in areas where fire blight is produced. In Republic of Korea, governors, researchers, and growers are aware of the problem caused by the emergence of resistant strains, fortunately, despite nationwide surveillance and routine monitoring, no reports of streptomycin-resistant strains have been made to date (Lee et al., 2018). In addition to the significance of streptomycin resistance, low efficacy during field treatment of streptomycin is also important. Because this phenomenon may arise from rebounding of pathogen via dormancy of pathogenic bacteria following streptomycin treatment at inappropriate concentrations, interval, and timing.

Dormancy has been reported to be one of the survival strategies used by non-sporulating bacteria in their surrounding environments, and it can be divided into two phenomena: persistence and viable but non-culturable (VBNC) states (Ayrapetyan et al., 2015, 2018; Martins et al., 2018; Zou et al., 2021). Persister cells have been found to reduce killing rate, which represents the biphasic kill curve after the bacterial cells are exposed to stresses such as antibiotics and then re-grown on media after the stresses are removed (Orman and Brynildsen, 2013). In contrast, VBNC cells, which are also described to be alive, lose their ability to grow on media with induced stresses such as antibiotics, but their re-growth requires some triggers such as carbon sources or in case of pathogens with natural hosts (Ayrapetyan et al., 2018). According to the mentioned concept between these two states, there seems to be a different strategy for bacteria to survive under unfavorable conditions. However, both persister and VBNC cells were formed under similar environmental stresses and stochastically (Fisher et al., 2017; Ayrapetyan et al., 2018), and no statistical differences were found between these two states of dormancy based on antibiotic tolerance, recovery rates, morphology, and metabolic activity (Kim et al., 2018a). Therefore, persister cells and VBNC state are likely to be part of a continuum of dormancy according to physiological relatedness (Ayrapetyan et al., 2015, 2018).

Although studies on plant pathogenic bacteria have focused more on VBNC than on persister cells, a few studies have shown that VBNC may survive under unfavorable conditions such as copper or chlorine exposure, low temperature, oxidative stress, and starvation (Ghezzi and Steck, 1999; Grey and Steck, 2001; Venisse et al., 2001; Ordax et al., 2006; Rodrigues et al., 2008; Álvarez et al., 2008; Santander et al., 2014; Postnikova et al., 2015; Santander and Biosca, 2017), and persister cells have only been reported for two bacteria (Muranaka et al., 2012; Peng et al., 2021). Thus, in this study, we examined the two dormancy states in *E. amylovora* (persistence and VBNC) after treating it with streptomycin, which is the most widely used bactericide for fire blight control in Republic of Korea. We also examined the relationships between these two states in terms of composition rates and periods, phenotypic properties, and appropriate treatment to reduce the survival strategies of *E. amylovora* cells that are in the persistent and VBNC state.

## Materials and methods

### Streptomycin-degradation test

Streptomycin-degradation tests were conducted using both hydrolysis and photolysis. In the hydrolysis test, streptomycin solutions were prepared in 50 mL conical tubes containing each 50 mg/mL of laboratory streptomycin (Streptomycin Sulfate, Fujifilm Wako Pure Chemical Co., Ltd. Osaka, Japan) and 250, 500, 1,000, and 2,500 μg/mL of bactericidal pesticide with streptomycin as 20% of active gradient (Agrepto, Kyungnong Co., Ltd, Seoul, Republic of Korea) and maintained at 28°C for 7 days in hydrolysis test. After 7 days, 10 μL of each streptomycin solution at 7 days was spot-inoculated on the center of mannitol glutamate yeast extract (MGY) medium that had been pre-plated with 100 μL of *E. amylovora* TS3128 bacterial suspension (Kang et al., 2021) at an optical density (OD) at 600 nm of 0.1 (approximately 3.1 × 10^8^ CFU/mL) and incubated at 28°C for 2 days. The effect of streptomycin degradation via hydrolysis was considered as an inhibitory zone against TS3128 by comparing the reduced size of the inhibitory zone with day 0 solution. In the photolysis test, 50 and 500 μg/mL of laboratory and bactericidal pesticide streptomycin solutions in 50 mL conical tubes, respectively, were exposed to an OD of 302 nm (UVB) using Ultraviolet Transilluminator (MaestroGen Inc., Hsinchu City, Taiwan) for 2 days, and TS3128 was then inoculated at a concentration of OD_600 *nm*_ = 0.3. The effect of UVB on streptomycin degradation was assessed by counting the colony forming unit (CFU/mL) of colonies grown on MGY medium without streptomycin at daily intervals until 5 days. All experiments were performed twice.

### Killing curve after treatment with streptomycin

To confirm whether *E. amylovora* TS3128 cells entered the persistent state, which represents the biphasic kill curve after streptomycin treatment, each single colony of TS3128 was cultured in King’s B broth at 28°C for 20 h and then sub-cultured in the same liquid medium at 28°C for 5, 20, and 96 h to correspond to lag, early stationary, and late stationary phase, respectively. At the sub-culture phase, 50 and 500 μg/mL of laboratory and pesticide streptomycin were added to each phase-culture, respectively, and was shaken at 28°C until 5 and 8 h for lag and early and late stationary phases, respectively. To remove residents of streptomycin, 1 mL of samples were collected twice an hour, re-suspended in 10 mM MgCl_2_ twice, and colonies were counted after 8 h on MGY agar medium. To date, a streptomycin resistant isolate of *E. amylovora* has not been found in Republic of Korea; therefore, a streptomycin resistant isolate of *Pseudomonas syringae* pv. *actinidiae* biovar 2, which was isolated in Republic of Korea, was used as a negative control (Psa2, Lee et al., 2020). Psa2 was cultured in King’s B broth with or without streptomycin for 20 h, and 1 mL of samples were collected and re-suspended with same procedures for TS3128. Colonies were then counted after 0, 12, and 24 h on King’s B medium with or without supplemented laboratory streptomycin (50 μg/mL).

To determine the end point of the persistence state induced by streptomycin, sub-cultivation was conducted in King’s B broth and distilled water, respectively. The CFUs of growing colonies were counted several times up to 72 h following previously described procedures. The experiments were conducted two times.

### Cells staining and flow cytometry

To prepare the early stationary phase during staining, *E. amylovora* cells were cultivated in King’s B broth at 28°C for 20 h as the nutrient rich case. The cultures were then centrifuged at 4,000 rpm for 6 min, re-suspended in 10 mM MgCl_2_ twice, and the pellets were suspended in same volume of distilled water as lack of nutrient case. Following the preparation of the two case samples, 500 μg/mL of pesticide streptomycin was added to two samples, and 1 mL of samples were taken at 3 days intervals till 9 and 15 days for nutrient rich and deficient status, respectively, and rinsed in 10 mM MgCl_2_ twice to remove streptomycin residue.

*E. amylovora* cells were stained with SYTO9 and propidium iodide (PI) using a BacLight Live/Dead viability kit (Invitrogen, CA, USA) to count total, live, and culturable cells according to previous reports (Santander et al., 2014; Kim et al., 2023). This was performed to produce a biphasic kill curve within the first few hours of cultivation, which showed a declining growth curve and identified the VBNC state after streptomycin treatment. To determine the proportions of each different physiological state in VBNC, staining with RedoxSensor Green (RSG) and PI was also conducted using the BacLight RSG Green vitality kit (Invitrogen, CA, USA) in accordance with a recommended protocol and previous report (Patel et al., 2021). Each 1 mL of the two case samples contained 1 μL of SYTO9 (3.34 μM) and PI (20 mM) or each of 1 μL RSG (1 mM) and PI and were incubated for 15 min in the dark. Subsequently, 200 μL of each stained samples were added to a FACS test tube and exposed for 1 min to FACSCalibur (BD Biosciences) and FACSymphony (BD Biosciences) for SYTO9 and PI and RSG and PI, respectively. FL1 530/30 bandpass filter for SYTO9 and FL3 670LP filter for PI in FACSCalibur and FITC (fluorescein isothiocyanate) 530/30 filter for RSG and PE 575/30 filter for PI in FACSymphony were used to capture fluorescence. Data analysis was conducted using the BD CellQuest Pro and BD FACSDiva software for Live/Dead and RSG kit, respectively. A total of nine fields were captured for fluorescence in three repeats. Four locations were identified among the recorded events (Supplementary Figure 1): PI-red (Q1), RSG and PI merge-yellow (Q2), no signal (Q3), and RSG-green signal (Q4). These zones were designated by 70% ethanol-killing, persistence and VBNC state, unstaining, and live cells, respectively. The CFUs of the culturable cells grown on MGY medium were counted as culturable cells in both staining.

### Secondary treatment experiments

To find alternative reagents that can shorten the duration of the persistence and VBNC state of *E. amylovora* cells induced by streptomycin, early stationary phase cells in distilled water were treated with 500 μg/mL of pesticide streptomycin and 10 mM of carbon sources such as glucose, sucrose, fructose, and sorbitol. One day after the first treatment, the second treatment with registered pesticides such as streptomycin (Agrepto), oxytetracycline (Sungbocycline, Sungbo Chemicals Co., Ltd., Republic of Korea), and tri-basic copper sulfate (Saebinna, Syngenta Co., Ltd., Republic of Korea), was carried out. The same procedures were used to assess the impact of secondary treatment on the reduction of the persistence and VBNC state induced by treated streptomycin alone.

### Antibiotic uptake assay

Streptomycin absorption was measured as the diameter of the growth inhibition zone of normal *E. amylovora* cells after they were extracted using *E. amylovora* persister cells (Chen et al., 2019). Briefly, 1 mL of persister cells induced by streptomycin with 10 mM carbon sources or second treatments with antibiotics were centrifuged at 4,000 rpm for 6 min, washed twice with 10 mM MgCl_2_ buffer, resuspended in 100 μL of cell wall digestion buffer [30 mM Tris–HCl (pH 8.0), 1 mM EDTA, 1 mg/mL lysozyme], and incubated at ambient temperature for 2 h. The pellets were frozen by treating them three times with liquid nitrogen. Subsequently, they were denatured for 10 min at 90°C using heat block and centrifuged at 12,000 rpm for 3 min to separate the debris and supernatant. A 10 μL supernatant was used to inoculate a spot in the middle of the pre-inoculated MGY medium with normal *E. amylovora* cells (100 μL of OD_600 *nm*_ = 0.1) and incubated at 28°C for 24 h. The diameter of cell growth inhibition zone was measured in two repeats.

### Recovery assays

An *in vitro* test was used to confirm that the persister and VBNC cells had recovered after the second antibiotic treatment following streptomycin treatment. Following the second treatment, 1 mL of cells were centrifuged at 4,000 rpm for 6 min and then washed twice in 10 mM MgCl_2_ buffer. Thereafter, 50 μL of cells was injected into 5 mL of M9 minimal broth supplemented with 40 mg/mL of asparagine, aspartate, glutamine, tryptophan, thiamine, threonine, histidine, and nicotinic acid and 1.5 mg/mL of succinate and pyruvate. The mixture was then culture at 28°C for 24 h in a shaking incubator. After plating 10 μL of the cultured cells onto MGY medium and incubating them at 28°C for 24 h, the number of bacterial colonies were counted as the number of recovered cells. Three plating cycles were used and three repetitions of recovery test were conducted. King’s B broth served as a positive control and was used in place of the M9 minimal broth supplemented nutrients.

### Statistical analysis

All experiments were independently repeated at least two times. Data were analyzed using *t*-test and analysis of variance, and the means were compared using Duncan’s least significant range test at *p* < 0.05. The analysis was performed using the Jamovi project, 2021 (ver. 2.3.21.0).

## Results

### Subpopulation of *E. amylovora* is not related to streptomycin degradation

The degradation rates of different concentration of streptomycin solutions in water at day 7 were not significantly affected, indicating that streptomycin degradation by hydrolysis was not significant (Supplementary Figure 1A). However, the inhibitory zone of 2,500 μg/mL of bactericidal pesticide at day 7 was significantly reduced compared to that at day 0. This result demonstrated that the inhibitory zones of bactericidal pesticide against *E. amylovora* emerged in a dosage-dependent manner, such that high concentrations (e.g., 2,500 μg/mL) might be degraded to reduce to inhibition size in water under normal light during the 28°C test periods. Streptomycin degradation by UVB, which is a strong factor in photolysis, was not exhibited under the tested conditions in this study (Supplementary Figure 1B). Therefore, we believe that the experimental system has the potential to determine whether *E. amylovora* can enter dormancy states, including persistence and VBNC, when treated with streptomycin.

### Streptomycin treatments produce *E. amylovora* persister cells

**FIGURE 1:**
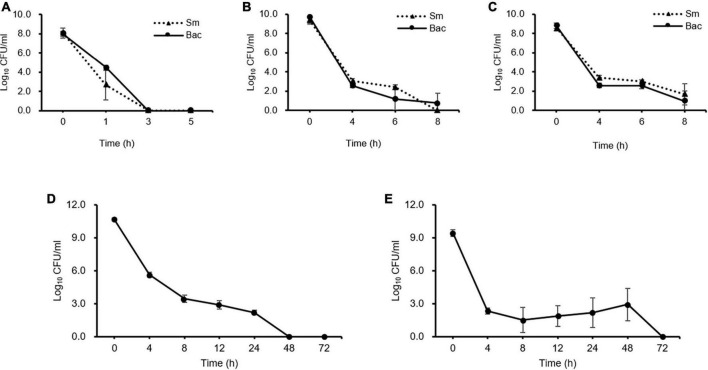
Comparison of persister cells after streptomycin treatments in *Erwinia amylovora* at 5 h for lag **(A)**, 20 h for early stationary **(B)**, and 96 h for late stationary **(C)** phases. *E. amylovora* cultures were treated with 50 and 100 mg/mL of laboratory and pesticide streptomycin, respectively, when the cells were inoculated in all three phases. The colony forming units were counted immediately after both streptomycin treatments and at 2 h intervals. Determination of the end point of the persister cells in *E. amylovora* in King’s B broth **(D)** and distilled water **(E)**. Data represent means of two replicates performed twice; error bars represent the standard deviation.

Kinetic killing curve assays were used to describe the subpopulation of *E. amylovora* TS3128 in the presence of 50 and 500 mg/mL concentrations of the laboratory and pesticide streptomycin. In the lag phase, the population declined within 3 h and most of them died (Figure 1A). In contrast, the number of survivors of early and late stationary phase reduced at 4 h and then remained the same until 8 h in both laboratory and pesticide streptomycin treatments (Figures 1B, C). These results suggested that both streptomycin treatments caused TS3128 cells in the stationary phase to enter the culturable persistence state. However, populations of streptomycin-resistant isolate (Psa2) did not decline when treated with streptomycin, and they showed a growth curve that was comparable to King’s B broth without streptomycin (Supplementary Figures 2A, B), indicating that Psa2 was not persistent. The end points of persistence in the kinetic killing curve of King’s B broth and distilled water were determined to be 24 and 48 h, respectively (Figures 1D, E), indicating that the combination of streptomycin treatment and lack of nutrient treatment may induce a longer period of persistence than just streptomycin treatment only.

### VBNC state is induced after inducing the persistence state by streptomycin treatment

**FIGURE 2:**
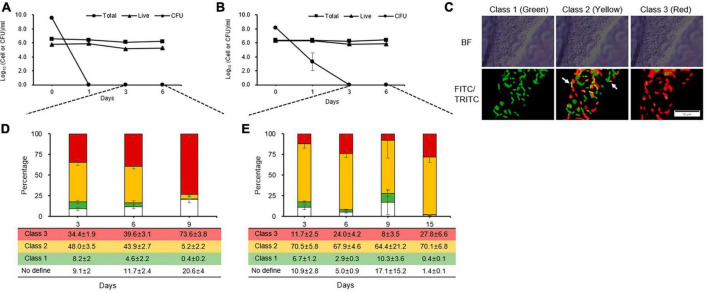
Distinct physiological states of unculturable *Erwinia amylovora* cells, presumable viable but non-culturable, after streptomycin treatment in King’s B broth **(A)** and distilled water **(B)**. Closed squares, triangles, and circles represent total, viable, and culturable cell count from early stationary phase with streptomycin treatment, respectively. **(C)** Representative cells depicting *E. amylovora* stained with RSG and PI classes used in this study. The ratio represents the total proportion of all cells in a class at King’s B broth **(D)** and distilled water **(E)** at different time point, as counted across nine images collected from three repetitions. Error bars represent the standard deviation across three independent experiments. BF, bright field; FITC/TRITC, merged captures between 488 and 532 nm wavelength for GFP and RFP, respectively. Scale bar = 10 mm.

During the counting of total, live, and culturable cells, culturable cells were observed until days 1 and 2 in King’s B broth and distilled water, respectively (Figures 2A, B). These patterns were linked to those of the kinetic killing curves, indicating that the persistence state occurred early in cells that had contact with streptomycin and would last longer under severe stress condition than in single stress. However, unculturable cells that were considered to be in the VBNC state after the persistence state were analyzed by staining each individual cell according to its physiological characteristics. We were unable to get the five categories that Patel et al. (2021) defined, because in this study, only two fluorescent dyes (the green vitality indicator RSG and red membrane permeability indicator PI) and one mixture fluorescence of them were used. Instead, we identified three classes (Figure 2C) and quadrant (Supplementary Figure 3): class 1, redox-active cells with intact membranes being regarded VBNC cells in this experiment (green, Q4 in FITC-A image); class 2, which are redox-active cells with damaged membranes and considered to be true VBNC cells (yellow, Q2 in FITC-A image); class 3, redox-inactive cells with damaged membranes (red, Q1 in FITC-A image); and no defined class, unstaining cells (white, Q3 in FITC-A image).

Up to 3 and 6 days after being treated with streptomycin in King’s B broth, an average of 34% and 39% of the populations were composed of class 3. After exposure to streptomycin at day 9, class 3 increased by an average of 73%, indicating that many populations changed to damaged membrane cells, which eventually became dead cells (Figure 2D). Unlike in King’s B broth, the populations VBNC cells in class 1 and 2 were the highest, with an average of 71, 70, 74, and 70% for 3, 6, 9, and 15 days, respectively, in streptomycin treated cells in distilled water (Figure 2E). However, class 3 populations did not significantly increase during the long exposure time to streptomycin. Thus, *E. amylovora* cells were maintained for longer periods as VBNC state in nutrient-deficient condition than in nutrient-rich one.

**FIGURE 3:**
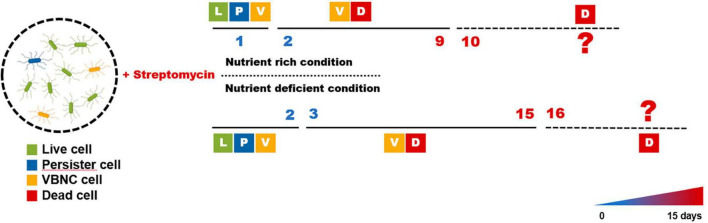
Predicted progression of the persister and viable but non-culturable (VBNC) cell formation in *Erwinia amylovora* after streptomycin treatment. Majority of the cells remained culturable after streptomycin treatment (called persister) for 1 and 2 days in King’s B broth and distilled water, respectively; however, few cells were presented as both live and VBNC state during those times. After each time point, bacterial cells were found to be in VBNC state, indicating that they lost culturability after continuous exposure to streptomycin. The membrane of all cells were affected after 10 and 15 days in nutrient rich and nutrient deficient conditions, respectively, to be considered dead cells.

These findings allowed *E. amylovora* to experience periods of persistence and VBNC states after exposure to streptomycin, as shown in Figure 3. The persister cells were visible until day 1 before losing their culturability and became VBNC state during days 1–9, and they may have become dead in nutrient-rich condition such as King’s B broth after that time. The persister cells could be seen for up to 2 days in nutrient-deficient conditions such as the distilled water used in this study. After 2 days, they varnished and seemed to be sustained to VBNC cells for an additional 15 days, and they may begin to die after this time. In both conditions, both the persister and VBNC cells were found at those duration times, suggesting that the persistence and VBNC states represent a continuum of dormancy that aids in preventing target activation of streptomycin.

### Sucrose and copper reduce streptomycin-induced persistence and VBNC state in *E. amylovora*

**FIGURE 4:**
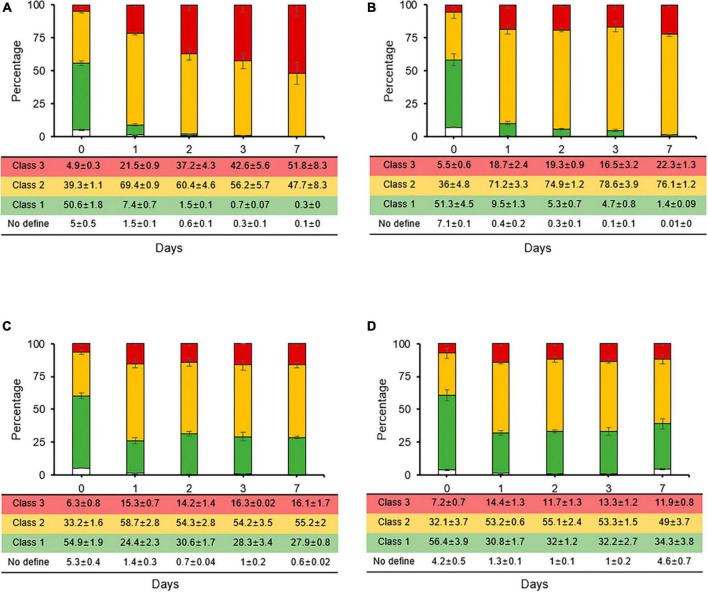
Relative proportions of *Erwinia amylovora* individuals in three physiological classes after streptomycin treatment with carbon sources such as sucrose **(A)**, glucose **(B)**, fructose **(C)**, and sorbitol **(D)**. Each ratio was calculated from nine fields with three repetitions. Error bars represent the standard deviation across three independent experiments.

The reduction rates of the persistence and VBNC states induced by streptomycin were evaluated through co-treatment cultivation with carbon sources and second treatment with chemical control agents for fire blight. When rates of stained cells were compared between streptomycin treatment and co-treatments with carbon sources, class 2 rate, which represents the persistence and VBNC state, decreased from 67% (Figure 2E) to 47% in sucrose co-treatment (Figure 4A). Conversely, the rates of class 3, which represents the dead cell caused by membrane damage, increased from 24% to a maximum of 51%, suggesting that sucrose is utilized to activate metabolism-related normal growth. The rates of stained cell types did not change significantly for glucose, fructose, and sorbitol (Figures 4B–D). However, in co-treatments of fructose and sorbitol, the rates of class 1 increased to a maximum of 27–34% at day 7, and populations of culturable cells increased significantly (data not shown), suggesting that these two carbon sources may have initiated re-growth. Thus, we concluded that glucose, fructose, and sorbitol should not be used in formulation of additive composites.

**FIGURE 5:**
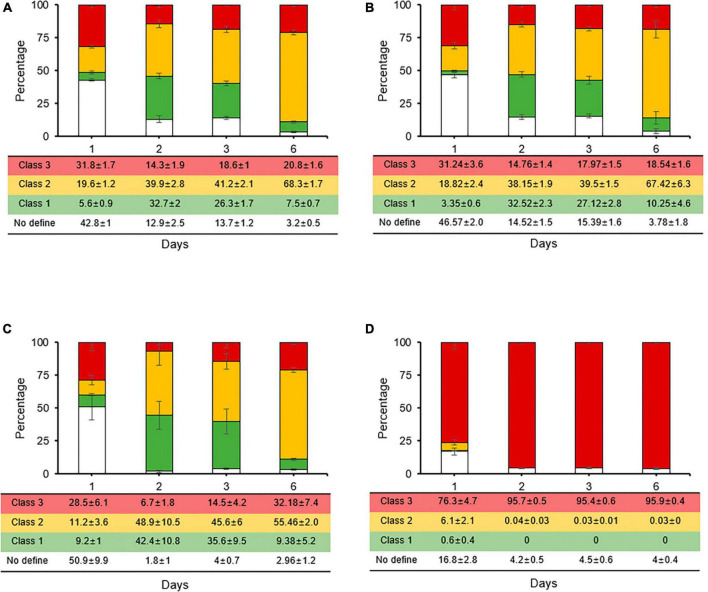
Relative proportions of *Erwinia amylovora* individuals in three physiological classes after single streptomycin treatment **(A)** following second treatment with streptomycin **(B)**, tetracycline **(C)**, and copper **(D)**. Each ratio was calculated from nine fields with three repeats. Error bars represent the standard deviation across three independent experiments.

The rates of the three classes in the second treatments of registered pesticides (streptomycin and oxytetracycline) all had the same ratios with streptomycin alone (Figures 5A–C), and the CFUs of culturable cells disappeared at day 3 (data not shown). These results suggest that the two most popular agriculture-antibiotics did not affect the persistence and VBNC states induced by first streptomycin treatment. However, all the types of stained cells quickly changed to class 3 in the second treatment with copper (Figure 5D); thus, we concluded that unculturable cells after 3 days with copper were not in the VBNC state but were all dead cells under *in vitro* condition. However, two or three treatments with different agricultural antibiotics are recommended as a control technique for fire blight under field conditions. Therefore, according to our results, copper should be used as a treatment once every three treatments.

In the streptomycin absorption experiment, co-treatment of the persister cells with sucrose showed larger inhibitory zone against normal *E. amylovora* growth than co-treatment of the persister cells with glucose, fructose, and sorbitol (Table 1), and this corresponded to the changed ratios of classes 2 and 3. However, the inhibitory zones of the second treatment of antibiotics were significantly smaller in size than those of the carbon source treatment (Table 1), indicating that antibiotics were not activated by proton motive force.

**TABLE 1:** Carbonsources enhance the uptake of streptomycin in streptomycin-induced *Erwinia amylovora* persister cells but not after the second antibiotic treatment.

Treatment		Days			
		**0**	**1**	**2**	**3**
Single treatment	Sm	0	0.35 ± 0.3	1.0 ± 0.1	1.11 ± 0.1
Co-treatment with carbon sources	Sucrose	0.75 ± 0.1	1.72 ± 0.1	UT^a^	1.45 ± 0.05
	Glucose	0.75 ± 0.2	1.32 ± 0.3	UT	1.43 ± 0.1
	Fructose	0.46 ± 0.4	1.41 ± 0.1	UT	1.61 ± 0.06
	Sorbitol	0.13 ± 0.2	1.12 ± 0.1	UT	1.72 ± 0.1
Second treatment	Sm	0	0.47 ± 0.5	0.83 ± 0.0	1.05 ± 0.1
	Oxytet	0.47 ± 0.5	0.31 ± 0.3	1.57 ± 0.2	1.62 ± 0.2
	Cop	0.13 ± 0.2	1.83 ± 0.1	1.95 ± 0.2	1.9 ± 0.1

Streptomycin uptake rate was measured based on the size of the inhibitory zone of streptomycin obtained from the cytoplasm of the persister cells. Data represent means ± standard deviation of three replicates in independent experiments with two repetitions. Sm (bactericidal streptomycin), Oxytet (bactericidal oxytetracycline), Cop (tri-basic copper sulfate), and UT (untested).

^a^UT indicates untested.

### Oxytetracycline eradicates streptomycin-induced persistence and VBNC state in *E. amylovora*

**FIGURE 6:**
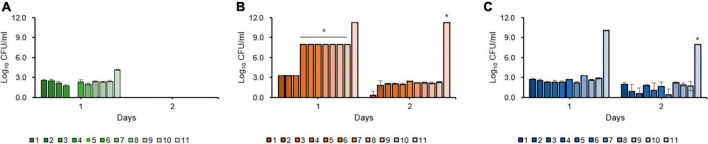
Resuscitation of persister and viable but non-culturable (VBNC) cells after single streptomycin treatment following second treatment with oxytetracycline **(A)**, single bactericidal streptomycin **(B)**, and second bactericidal streptomycin treatment **(C)**. Oxytetracycline inhibits the formation and maintenance of the persister and VBNC cells of *Erwinia amylovora* induced by streptomycin in all tested amino acids and metabolites. Error bars represent the standard deviation across three independent experiments. *Indicates recovered colonies with each amino acids and metabolites; however, CFUs were not counted due to countless numbers.

No change in the ratios of classes 2 and 3 in the second antibiotic treatment was observed, and persister cells of the secondary oxytetracycline treatment did not recover after 2 days (Figure 6A) compared to bactericidal streptomycin treatment alone (Figure 6B) and secondary bactericidal streptomycin treatment (Figure 6C), regardless of the types of amino acid and metabolites. Conversely, the recovered cells from the second streptomycin treatment showed comparable CFUs, indicating that the persister cells induced by single streptomycin treatment were not eliminated by second streptomycin treatment (data now shown). Thus, we concluded that streptomycin persister cells did not survive in the presence of oxytetracycline and oxytetracycline treatment because the second application was highly effective in eliminating culturable streptomycin persister cells.

## Discussion

Regardless of the two- or three-times treatment strategies in pre- or post-blooming seasons, streptomycin remains an important component for controlling fire blight in Republic of Korea (RDA’s standard operating procedure (SOP) for fire blight control practice in Republic of Korea). According to the SOP, streptomycin and oxolinic acid should be applied using a two times strategy, and oxytetracycline, streptomycin, and oxolinic acid should be applied using a three times strategy. This suggest that streptomycin is a key component of chemical control strategy. However, because fire blight is still classified as a quarantine disease, any efficacy tests against this disease must be conducted by people certified to handle quarantine pathogens in a confined and insulated greenhouse called plant pest containment facility (Level 3), which we do not yet have. Thus, the effectiveness of their consecutive applications could not be guaranteed owing to the small scale of experimental designs and repetitions and the possibility of excessive subjectivity in evaluation of each test. Furthermore, in a few cases with copper (Ordax et al., 2006) but not in a majority of cases with antibiotics, results of the fire blight pathogen *E. amylovora* varied by dormancy, including the induction of the persister and VBNC cells by chemical control agents. Recently, Patel et al. (2021) reported that *P. syringae* pv. *phaseolicola* has the persistence to survive under streptomycin treatment. However, there is no study on the persistence and VBNC states in fire blight pathogen treated with streptomycin. Therefore, the present study aimed to identify the durations and ratios of the persistence and VBNC states that streptomycin induces in *E. amylovora* and the agents that eliminate these two states.

To achieve these, we first verified whether streptomycin was degraded by hydrolysis and photolysis during the test period. In a previous report, streptomycin lost its antibacterial activity due to degradation, and it was faster under light exposure water than it did in the dark water (Shen et al., 2017). However, in this study, throughout the measured durations for 7 and 5 days in water and UVB exposure, respectively, neither laboratory nor pesticide streptomycin was degraded in water under normal light at 28°C. This result showed that photo-degradation of kasugamycin and oxytetracycline had negative effects on their activities, but that of streptomycin did not have any negative impact on its activity (Slack et al., 2021). Thus, without the use of artificial streptomycin-degradable components, we could evaluate subsequent experiments, in which *E. amylovora* is exposed to streptomycin, to induce dormancy states.

Several important evidence for persistence differentiation between resistance and tolerance against antibiotics is the biphasic killing curve have been reported in the literature (Brauner et al., 2016; Renbarger et al., 2017; Balaban et al., 2019). In the early and late stationary phases, *E. amylovora* cells exhibited the two phases of the decline curve, that is, a rapid decline at first and a gradual decline at second with general concentrations of both laboratory and pesticide streptomycin, but this was not observed in the lag phase. Previous reports have shown that persistence is not logarithmic phase, and stationary phase cells have significantly higher numbers of the persistence cells than log phase cells (Ayrapetyan et al., 2018; Patel et al., 2021). Hence, early stationary phase studies were conducted in subsequent experiments. In addition, Psa2, the streptomycin-resistant strain that was isolated in Republic of Korea (Lee et al., 2018) and used as the negative control in this study, was able to replicate and survive, but *E. amylovora* was not able to replicate and survive. This means that *E. amylovora* cells were exposed to stress from streptomycin and were able to persist under stressful conditions.

The culturable cells were counted until 24 and 48 h in King’s B broth and distilled water using extended biphasic kill curve experiments and a BacLight Live/Dead viability kit. It means that persistent and living culturable cells would be damage or die from continuous exposure to streptomycin. Thus, the persister cells, which are culturable on solid media, seemed to change to VBNC as a result of continuous exposure to streptomycin until they are doomed to die. This suggests that persistence is pre-status before changing to VBNC and a continuum phenotype against stress such as streptomycin treatment. The VBNC cells are generally known to be in a deeper state than the persister cells (Ayrapetyan et al., 2018). In addition, following streptomycin treatment, *E. amylovora* cells were shown to exhibit persistence rather than tolerance because single cell staining ratios revealed three classes of subpopulations based on redox-activation and membrane stability. Generally, persistence affects only a subpopulation of cells under stress (Balaban et al., 2019); thus, it is clearly demonstrated that streptomycin treatment caused *E. amylovora* cells to become persistence rather than developing resistance to the antibiotics. Actually, single cell staining using PI and RSG, which are membrane permeability and redox indicators related to metabolic activity, respectively (Baert et al., 2016), in the Live/Dead viability kit and RSG Green vitality kit are very powerful tools to show status at single cell. Single cell observation using flow cytometry after staining indicate that all distinct cell statuses survive until culturable stage, and the administration of streptomycin causes a prolonged increase in membrane-damaged cells. These findings are consistent with previous reports that staining can confirm the presence of persister and VBNC cells in *Escherichia coli* (Kim et al., 2018b) and that the number of the VBNC cells is higher than that of the persister cells after antibiotic treatment (Bamford et al., 2017).

In addition, cell types and their up and down variations can be quantified using single cell staining with flow cytometry to identify second treatment agents that reduce the persister and VBNC cells after the initial streptomycin treatment. In previous reports involving environmentally relevant bacteria, glucose, mannitol, fructose, and pyruvate enabled the gentamicin killing of *E. coli* and *Vibrio cholera* persister cells (Allison et al., 2011; Paranjape and Shashidhar, 2020). *E. coli* persister cells use glycerol mostly as carbon sources (Amato et al., 2014), suggesting that increased antibiotic killing effects are stimulated by proton motive force after increased antibiotic uptake that was responsible for the persister cell killing (Orman and Brynildsen, 2013). Among the carbon sources investigated in this study, glucose and sucrose showed the greatest killing effect on *E. amylovora* persister cells induced by streptomycin. In addition, streptomycin potentiation by carbon sources in *E. amylovora* revealed an increase in inhibitory areas after carbon source treatment in comparison to single streptomycin treatment. This suggests that the increased uptake was responsible for the killing of the persister cells in *E. amylovora*. Hence, *E. amylovora* persister cells might be mostly eradicated in a more practical way using sucrose and then glucose in a metabolism-dependent manner.

To determine the most effective strategy for eradicating streptomycin persister cells using single cell observations in a real-world control system with antibiotics, we discovered that copper can completely eliminate persister cells that exhibit classes 1 and 2 that changed to class 3. This pattern was also described in *Xanthomonas citri* persister cells induced by antibiotics, high temperature, and metals (Martins et al., 2021). However, coppers were known agricultural agents with negative effects, such as injury on immature shoots and fruits on apple trees; hence, farmers prefer not to use them at any growing stage of apple trees. Thus, practical approaches are needed to define the usage of coppers in preventing injury and eliminating persister cells. In addition to the copper effect, second treatments with either streptomycin or tetracycline did not change the classes deduced by flow cytometry when compared to a single streptomycin treatment, indicating that neither of the antibiotic treatments were effective in eliminating the persister cells induced by streptomycin. Additionally, the diameters of the inhibitory zones were almost similar between the single streptomycin and second streptomycin or tetracycline treatments. This indicates that combined treatments of antibiotics, particularly aminoglycoside and tetracycline, did not increase the absorption of antibiotic by stimulating proton motive force. It is believed that antibiotics never used nutrients and growth factors to increase uptake of streptomycin. However, in the recovery experiments, *E. amylovora* persister cells completely lost their culturability on solid medium, suggesting that tetracycline has the strongest effect in eliminating the persister cells induced by streptomycin in a manner that is different from the metabolism-independent method. Hence, streptomycin treatment, with sucrose as supplement or additive agent in the final formulation, is recommended in orchards to have optimal result in field settings. Tetracycline should also be used in combination with other treatments rather than streptomycin alone to treat fire blight more effectively.

## Data availability statement

The original contributions presented in the study are included in the article/Supplementary material, further inquiries can be directed to the corresponding author.

## Author contributions

YK: Writing – original draft, Conceptualization, Data curation, Formal Analysis, Methodology, Validation. HC: Formal Analysis, Data curation, Methodology, Writing – original draft. DP: Writing – original draft, Writing – review and editing, Conceptualization, Data curation, Funding acquisition, Resources, Supervision.
