# Supplementary material for: Persistence and viable but non-culturable state induced by streptomycin in Erwinia amylovora
Source: Front Microbiol. 2024 Feb 21;15:1346300. doi: 10.3389/fmicb.2024.1346300 (PMC10914980; doi:10.3389/fmicb.2024.1346300)
Supplement: Supplementary file 1 [file Data_Sheet_1.docx]

Supplementary Material

# Supplementary Figures


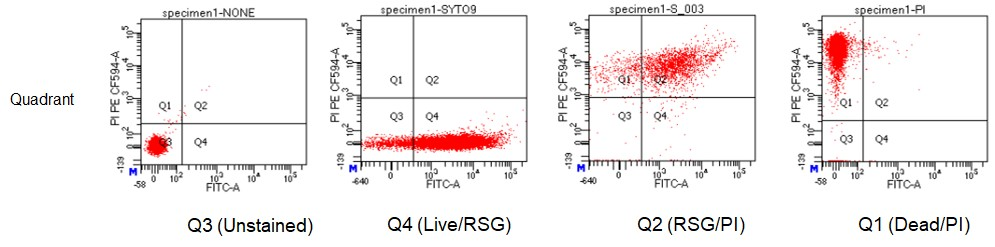


**Supplementary Figure 1**. Representative density dot plots of RSG and PI-stained *Erwinia amylovora* cells after streptomycin treatment. Quadrant showed Q1 (PI-red), Q2 (RSG and PI merge-yellow), Q3 (unstained), and Q4 (RSG-green). Each population in quadrant were counted, and all cells were sorted into FACS tube for 1 min.


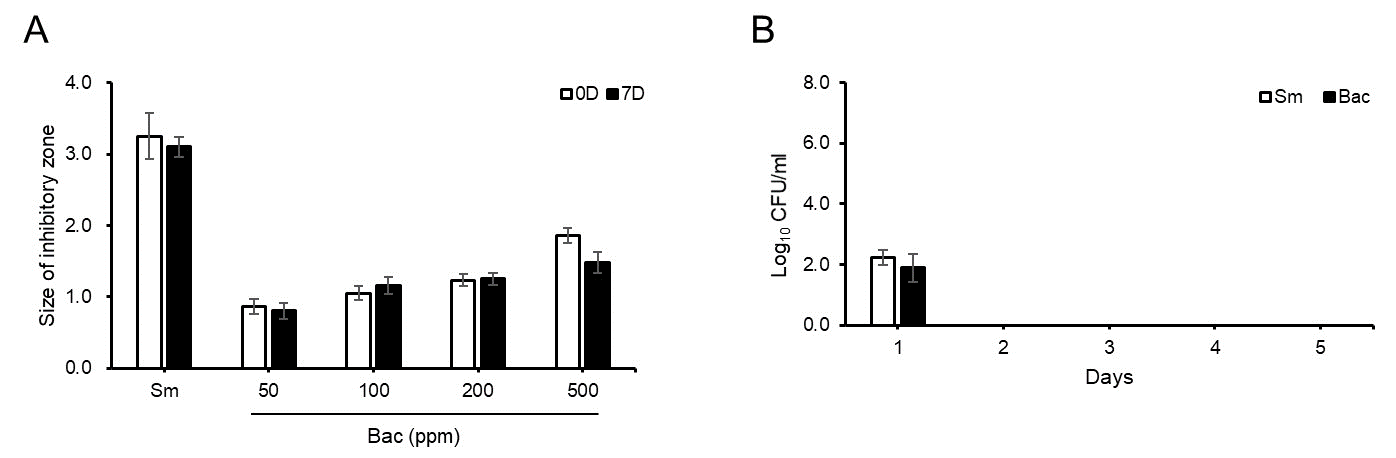
 **Supplementary Figure 2**. Rate of streptomycin degradation based on hydrolysis (A) and photolysis (B). Hydrolysis and photolysis tests were conducted in distilled water for seven days and exposed to UV 302 nm for two days, respectively. Data represent means of three replicates performed twice; error bars represent the standard deviation. Sm: laboratory streptomycin. Bac: bactericidal pesticide streptomycin.


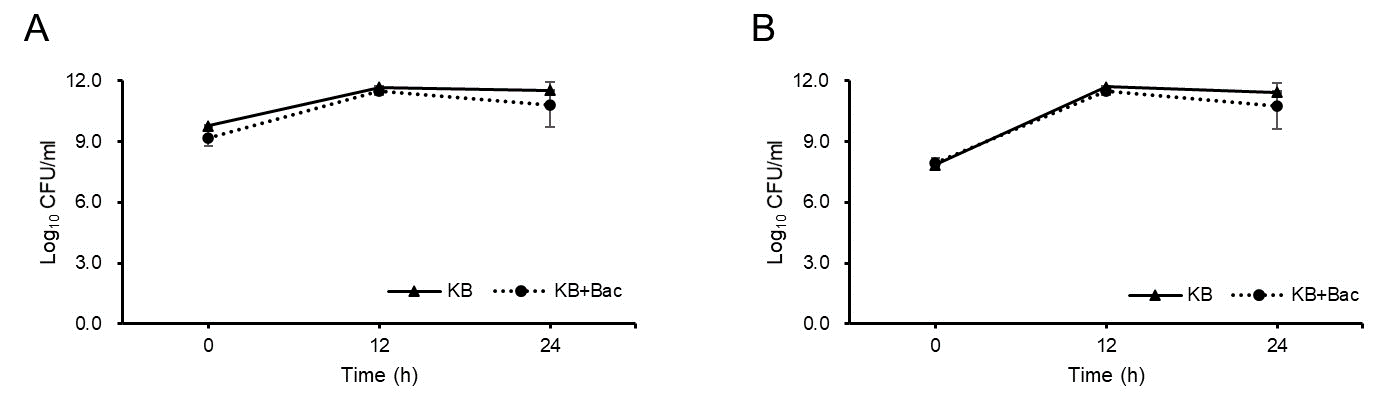
 **Supplementary Figure 3**. Population of streptomycin resistant isolate of *Pseudomonas syringae* pv. *actinidiae* biovar 2 (Psa2) in King’s B broth in the presence or absence of bactericidal pesticide streptomycin. CFUs count from plate on King’s B medium (A) and King’s B medium supplemented with streptomycin (B). Data represent means of three replicates performed twice; error bars represent the standard deviation. Bac: bactericidal pesticide streptomycin.
